# Associations of hemoglobin levels with structural knee MRI findings at 33 years of age in a general population-based birth cohort

**DOI:** 10.1016/j.ocarto.2026.100841

**Published:** 2026-06-12

**Authors:** Atte Tapiola, Joona Tapio, Antti Kemppainen, Miika T. Nieminen, Simo Saarakkala, Mika T. Nevalainen, Peppi Koivunen

**Affiliations:** a Research Unit of ECM and Hypoxia, Faculty of Biochemistry and Molecular Medicine, University of Oulu, P.O. Box 5400, FIN-90014 Oulu, Finland; b Biocenter Oulu, University of Oulu, P.O. Box 5400, FIN-90014 Oulu, Finland; c Research Unit of Health Sciences and Technology, Faculty of Medicine, University of Oulu, P.O. Box 5000, FI-90014 Oulu, Finland; d Medical Research Center Oulu, University of Oulu and Oulu University Hospital Oulu, Finland

**Keywords:** Hemoglobin, Cohort studies, Knee joint/diagnostic imaging, Magnetic resonance imaging, Osteoarthritis

## Abstract

**Objective:**

To evaluate potential associations of hemoglobin (Hb) levels and knee magnetic resonance imaging (MRI) findings at 33 years of age in a subpopulation of the Northern Finland Birth Cohort 1986.

**Method:**

Subjects with available knee MRI, clinical and questionnaire data (*n* = 288) and Hb levels within the Finnish reference values, were included for a final study population of 275 subjects (40.7% males). The MRI Osteoarthritis Knee Score (MOAKS) was used for MRI grading. Imaging findings were evaluated in Hb tertiles in descriptive statistics and using Hb levels as a continuous variable in regression models.

**Results:**

Subjects in the high Hb tertile had higher BMI, fasting glucose, LDL cholesterol and blood pressure than subjects in the low Hb tertile. Any tibiofemoral and patellofemoral cartilage lesions were found in 23.0% and 52.6 % of joints in the low Hb tertile and 31.9% and 66.7% in the high Hb tertile. In contrast, any tibiofemoral osteophytes were detected in 21.0 % of joints in the low Hb tertile compared to 8.8% the high Hb tertile. Any knee effusion was more common in the high Hb tertile (54.9%) compared to the low Hb tertile (42.0%). Advanced osteoarthritic MRI findings were rare. In regression models, observed RRs were small. Hb levels were positively associated with any cartilage loss and negatively with tibial and femoral osteophytes.

**Conclusion:**

In this relatively symptomless cohort, Hb levels were not consistently associated with knee MRI findings. However, small cartilage lesions were more common in subjects with higher Hb levels.

## 1 Introduction

Hemoglobin (Hb) is the primary carrier of oxygen in the circulatory system, and its levels govern tissue oxygenation status [1]. Lower Hb levels within the normal range have previously been associated with a leaner body composition, the association being mediated by lesser tissue oxygenation and activation of the principal cellular response to hypoxia, the hypoxia-inducible factor (HIF) pathway [[2], [3], [4], [5], [6]]. The HIF pathway connects oxygenation status with gene expression and is a central regulator of energy metabolism, angiogenesis, inflammatory responses and extracellular matrix (ECM) homeostasis [7].

Osteoarthritis (OA) is a common degenerative joint disease affecting hundreds of millions of people worldwide. Knee joints are the most common site affected by OA [8]. MRI (MRI) is the standard of OA research over radiographs for identifying structural intra- and extra-articular changes [9,10]. As MRI features of OA are relatively infrequent and often mild in asymptomatic adults under 40 years of age [11], studies on subjects without obvious OA risk factors or previous injuries are sparse.

OA pathology is driven by the destruction of articular cartilage, mainly caused by the decomposition of ECM and death of chondrocytes [12]. Articular cartilage itself is a hypoxic environment and studies conducted *in vitro* suggest that HIF1α protects chondrocytes from apoptosis, increases ECM gene expression and cell viability [13]. Studies from human synovial fluid suggest HIF1α levels are related to the severity and progression of OA [14]. Systemic inflammation has been related to progression of OA earlier and it has been proposed to be a pathological driver of OA rather than a secondary symptom [15,16]. In experimental *in vivo* studies, HIF1α has been shown to have an attenuating effect on inflammatory signaling [17].

As cellular oxygen sensing mechanisms might be linked to OA, and higher Hb levels have been associated with heavier body composition, a key risk factor of OA already in the age of 16 years [18], we set out to study whether Hb levels are associated with OA-related MRI findings in young adults. The aim was to examine relations between Hb levels and key imaging findings in relatively healthy and mostly asymptomatic young adults.

## 2 Methods

### 2.1 Study population

The data for this study were derived from the Northern Finland Birth Cohort 1986 (NFBC1986) consisting of 99 % of individuals who were due to be born in the two northernmost provinces of Finland between July 1985 and June 1986 (*n* = 9432) [19]. The latest follow-up was at the age of 33 years. Knee MRI was conducted on 297 cohort members as a random subsample with no other selection criteria. 9 MRIs were incomplete and subsequently excluded. The knee joint reported subjectively as more symptomatic or the dominant knee in lack of symptoms (77.1% right, 22.9% left) was imaged [20].

A flow-chart of the study setup and population is presented in Fig. S1. The inclusion criteria for the current study were availability of (i) a completed knee MRI, (ii) clinical and postal questionnaire data at 33 years of age and (iii) Hb levels within the Finnish reference values (117–155 g/L for females and 134–167 g/L for males), thus representing an age-correlated 2.5%–97.5% of the Hb range [21]. The final population included 275 subjects (112 males and 163 females) with no missing data of any covariate (Table S1).

The NFBC1986 study was conducted according to the declaration of Helsinki and approved by the ethical committee of the Northern Ostrobothnia Hospital District in Oulu, Finland. All participants provided a written statement of informed consent at the time of the original NFBC1986 study visit. The authors obtained permission for this study from the NFBC (project number P1167). The study adheres to the STROBE guidelines.

### 2.2 Background information

Background information was determined by a postal questionnaire. Participants were dichotomized as ‘no’ or ‘yes’ regarding family history of knee OA (parents, siblings and grandparents) and prior lower limb fractures. Smoking status was classified as ‘never’ or ‘ever’ smokers. Physical activity score was determined as the sum of four questions assessing the duration (none = 0 to > 1.5 h = 6) and frequency (once a month at most = 0 to daily = 6) of exercise.

Right before the knee MRIs, the visual analogue version of The Western Ontario and McMaster Universities Osteoarthritis Index [22,23] scale (WOMAC) was used for assessing pain, stiffness and function of the imaged knee with scores recorded on a 0–10 visual analogue version for each question [8].

### 2.3 Clinical examinations and laboratory analyses

Participants were instructed to abstain from caffeine and smoking before the study visit. Trained nurses measured body height, weight, waist and hip circumference, brachial systolic and diastolic blood pressure (bp), and heart rate, and body mass index (BMI) was calculated.

Blood samples collected after overnight fasting were analyzed at NordLab Oulu. Blood Hb levels and fasting plasma glucose were determined with photometric and enzymatic assays. Photometric direct enzymatic measurement was used for determining low-density lipoprotein (LDL) cholesterol. High-sensitivity C-reactive protein (hs-CRP) was quantified with an immune nephelometric assay (BN ProSpec, Siemens Healthcare Diagnostics INC., Newark, DE).

### 2.4 MRI

Knee MRIs and their semi-quantitative assessments were performed according to previously detailed methods. Briefly, a single knee joint was scanned for every participant using a 3T MRI scanner with uniform imaging protocols: turbo spin echo T2 sequence with fat saturation, turbo spin echo proton density weighted sequence and double echo steady state sequence [8] (Table S3). Images were evaluated using the MRI Osteoarthritis Knee Score (MOAKS) system [24,25]. A board-certified radiologist and musculoskeletal radiology fellow with six years of training, following calibration sessions with a board-certified, fellowship-trained musculoskeletal radiologist (MTNe), with over a decade of experience, performed the scoring. For the current study, Hoffa-synovitis was also evaluated as either normal (0); mild (1); moderate (2) or severe (3) by and musculoskeletal radiology fellow. All MRI features of MOAKS except the number of subregional BMLs, synovitis - effusion, meniscal extrusion and hypertrophy were included in this study.

For analyses, MOAKS subregions were pooled into seven anatomical regions as described [8]: 1) tibial medial, 2) tibial lateral, 3) femoral medial, 4) femoral lateral, 5) tibiofemoral (regions 1–4 combined), 6) patellofemoral, and 7) any. For each anatomical region, the single highest MOAKS severity grade of its comprising subregions was used in analyses of cartilage loss, full-thickness cartilage loss, bone marrow lesion size, their associated subarticular bone marrow cysts and osteophyte size.

### 2.5 Statistical analysis

The population was first divided into sex-specific Hb level tertiles (low, medium and high; Fig. S1), which were then pooled to form Hb tertiles representing both sexes (Fig. S1). Background and clinical characteristics were evaluated across the study population and non-included participants (Table S1) and across the Hb tertiles (Table 1) in descriptive statistics. Continuous variables were checked for skewness and presented between Hb tertiles as mean (M) and standard deviation if normally distributed, or median (Mn) and interquartile range if non-normally distributed. Categorical variables were presented between Hb tertiles as counts (n) and percentages.

**Table 1:** **Background and clinical characteristics of the study population in Hb tertiles.** Data is presented as mean (M) and standard deviation (SD) for normally-distributed continuous variables, median (Mn) and interquartile range (IQR) for skewed continuous variables and count (n) and percentage (%) for count variables. M, mean; SD, standard deviation; OA, osteoarthritis; WOMAC, The Western Ontario and McMaster Universities Osteoarthritis Index scale; Hb, hemoglobin; BMI, body mass index; fP, fasted plasma; HDL, high-density lipoprotein; LDL, low-density lipoprotein; hs-CRP, high-sensitivity C-reactive protein.

	Low Hb (n = 100)	Medium Hb (n = 84)	High Hb (n = 91)
	M (SD)/Mn(IQR) or n (%)	M (SD)/Mn(IQR) or n (%)	M (SD)/Mn(IQR) or n (%)
**All participants**			
Males n (%)	43 (43.0)	32 (38.1)	37 (40.7)
Females n (%)	57 (57.0)	52 (61.9)	54 (59.3)
Age (years)	33.7 (0.4)	33.6 (0.4)	33.7 (0.4)
Prior lower limb fracture n (%)	11 (11.0)	9 (10.7)	13 (14.3)
Family history of knee OA n (%)	29 (29.0)	30 (35.7)	33 (36.3)
Never smoker n (%)	41 (41.0)	23 (27.4)	39 (42.9)
Physical activity score	15.2 (3.1)	14.5 (3.3)	14.8 (3.6)
WOMAC total (VAS-scale)	7.9 (18.5)	8.8 (11.8)	8.7 (16.5)
Hb (g/L)	130.0 (9.2)	136.7 (8.9)	145.8 (9.7)
BMI (kg/m^2^)	25.4 (4.6)	26.4 (4.6)	25.7 (4.5)
Waist circumference (cm)	85.8 (11.9)	88.8 (12.5)	88.1 (12.2)
Systolic blood pressure (mmHg)	112.13 (12.0)	111.4 (11.1)	113.4 (13.5)
Diastolic blood pressure (mmHg)	73.4 (9.2)	73.8 (7.6)	76.5 (9.3)
fP-glucose (mmol/L)	4.9 (0.3)	5.0 (0.4)	5.1 (0.9)
fP-LDL cholesterol (mmol/L)	2.7 (0.8)	2.8 (0.7)	2.9 (0.9)
Hs-CRP (mg/L)	0.8 (0.3–1.6)	0.7 (0.4–1.8)	0.7 (0.4–1.3)
**Males**			
Age (years)	33.7 (0.4)	33.6 (0.4)	33.7 (0.4)
Prior lower limb fracture n (%)	7 (16.3)	6 (18.8)	7 (18.9)
Family history of knee OA n (%)	12 (27.9)	15 (46.9)	10 (27.0)
Never smoker n (%)	17 (39.5)	7 (21.9)	13 (35.1)
Physical activity score	15.2 (3.5)	15.3 (3.3)	13.9 (4.0)
WOMAC total (VAS-scale)	6.6 (17.7)	6.2 (8.3)	8.6 (21.6)
Hb (g/L)	139.8 (4.4)	147.8 (1.4)	156.4 (3.9)
BMI (kg/m^2^)	24.6 (3.0)	26.2 (3.3)	26.4 (4.3)
Waist circumference (cm)	88.0 (8.6)	93.3 (9.3)	94.9 (10.8)
Systolic blood pressure (mmHg)	117.5 (9.0)	118.5 (8.8)	124.5 (11.9)
Diastolic blood pressure (mmHg)	73.8 (6.9)	75.2 (6.1)	80.8 (10.0)
fP-glucose (mmol/L)	5.1 (0.3)	5.1 (0.4)	5.3 (0.4)
fP-LDL cholesterol (mmol/L)	2.9 (0.7)	3.1 (0.7)	3.2 (0.9)
Hs-CRP (mg/L)	0.4 (0.2–0.9)	0.6 (0.3–1.0)	0.6 (0.2–1.0)
**Females**			
Age (years)	33.7 (0.4)	33.6 (0.3)	33.8 (0.3)
Prior lower limb fracture n (%)	4 (7.0)	3 (5.8)	6 (11.1)
Family history of knee OA n (%)	17 (29.8)	15 (28.8)	23 (42.6)
Never smoker n (%)	24 (42.1)	16 (30.8)	26 (48.1)
Physical activity score	15.2 (2.8)	14.0 (3.1)	15.4 (3.1)
WOMAC total (VAS-scale)	8.9 (19.2)	10.4 (13.2)	8.9 (12.2)
Hb (g/L)	122.7 (2.6)	129.9 (2.0)	138.5 (4.1)
BMI (kg/m^2^)	26.0 (5.5)	26.5 (5.3)	25.2 (4.6)
Waist circumference (cm)	84.2 (13.8)	86.0 (13.5)	83.4 (10.9)
Systolic blood pressure (mmHg)	108.1 (12.4)	107.0 (10.2)	105.8 (8.2)
Diastolic blood pressure (mmHg)	73.0 (10.7)	72.9 (8.3)	73.5 (7.7)
fP-glucose (mmol/L)	4.8 (0.3)	4.9 (0.4)	4.9 (1.1)
fP-LDL cholesterol (mmol/L)	2.5 (0.82)	2.6 (0.61)	2.7 (0.84)
Hs-CRP (mg/L)	1.0 (0.5–2.0)	0.8 (0.5–2.9)	0.9 (0.4–1.6)

Counts (n) and percentages of the most severe in-patient cartilage lesions, BMLs and osteophytes in the tibiofemoral and patellofemoral joint regions and meniscal morphology features and other MRI features were evaluated across the Hb tertiles for all participants, and males and females separately.

MRI features described above with <5 cases were not analyzed in regression models. For parameters with ≥5 cases, unadjusted regression analyses evaluating individual associations between Hb as a continuous variable (per 1 g/L increase) and individual knee MRI features were conducted. In case of a binary outcome a logistic regression model was used, and a Poisson regression model with robust standard errors was used for multi-classed outcomes. Two distinct multivariable regression models were also analyzed. The first model was adjusted for sex (Model 1) and the second model for sex and BMI (Model 2). In the models, the risk ratios (RRs) are given for 1 g/l Hb.

All the statistical analyses were performed using IBM SPSS statistics, version 29.0.0.0 (IBM Corp, Armonk, NY).

## 3 Results

### 3.1 Background and clinical characteristics of the study population

The background and clinical characteristics across Hb tertiles are presented in Table 1. All three tertiles had similar fractions of male and female participants of 33 years of age. The fraction of subjects with prior lower limb fractures were similar across the tertiles (10.7–14.3%). Having a family history of knee OA varied slightly between the tertiles, with slightly higher prevalence in the medium (35.7%) and high Hb tertiles (36.3%) compared with the low Hb tertile (29.0%). The medium Hb tertile had the lowest amount of never smoker subjects. No major differences in physical activity or WOMAC scores, which were overall low, were observed between the Hb tertiles.

Of clinical measures, some minor differences were observed across the Hb tertiles (Table 1). Subjects in the medium and high Hb tertiles had similar and higher BMI and waist circumference than subjects in the low Hb tertile. Fasting glucose and LDL cholesterol levels increased slightly according to Hb tertiles. Subjects in the high Hb tertile also had the highest systolic and diastolic bp. No clear differences were observed in hs-CRP levels between the Hb tertiles.

In sex-specific analyses, in males, BMI increased modestly but gradually from low tothe high Hb tertile, and similar trends were observed in waist circumference, systolic and diastolic bp, fp-glucose and LDL cholesterol. In females, prior fractures (11.1 %) and a family history of knee OA (42.6 %) were slightly more common in the high Hb tertile compared to other tertiles. No associations for BMI, waist circumference or bp, such as those observed in males, were detected in females, but a slight increase in LDL cholesterol was observed according to increase in Hb tertiles.

The background and clinical characteristics of included and non-included participants are presented in Table S1. No statistically significant differences were observed in background or clinical parameters. When observed in Hb tertiles, non-included participants showed a dose dependent increase in BMI, waist circumference, hs-CRP (Table S2) which was not seen in the study population (Table 1).

### 3.2 Counts of the most severe in-patient MRI-detected cartilage lesions, BMLs and osteophytes in the tibiofemoral and patellofemoral joint regions according to Hb tertiles

The distribution of the most severe cartilage lesions, BMLs and osteophytes across Hb tertiles for the whole study population, and males and females separately, are presented in Tables 2, S4 and S5, respectively.

In the medial and lateral tibiofemoral joint, the percentage of anatomical regions with no cartilage lesions ranged between 80.7% and 98.2% in the low Hb tertile and 75.9% and 94.4% in the high Hb tertile (Table 2). The greatest between low-to-high Hb tertile-differences in cartilage loss were observed in the medial femoral compartment (no cartilage loss in 80.7% and 75.9% of joints, respectively) and in the lateral femoral compartment (no cartilage loss in 98.2% and 92.6% of joints, respectively) (Table 2). In total, tibiofemoral cartilage lesions were found in 23.0% of the joints in the low Hb tertile and 31.9% in the high Hb tertile (Table 2). Most of the lesions were small and full-thickness cartilage lesions and BMLs were rare (Table 2). Most tibiofemoral osteophytes were small or doubtful, whereas medium and large osteophytes were infrequent (Table 2). In contrast to cartilage loss, tibiofemoral osteophytes were more common in the low Hb tertile (Table 2), with no osteophytes in 79.0% of the joints in the low Hb tertile compared to 91.2% in the high Hb tertile (Table 2). Similar distributions were observed for both males and females (Tables S4 and S5).

**Table 2:** **Counts of the most severe in-patient MRI-detected cartilage lesions, BMLs and osteophytes in the tibiofemoral and patellofemoral joint regions according to Hb tertiles.** Data is presented as count (n) and percentage (%). Hb, hemoglobin; FT, full thickness; BML, bone marrow lesion.

Hb	Tibial medial			Tibial lateral			Femoral medial			Femoral lateral			Tibiofemoral			Patellofemoral		
	Low Hb	Med Hb	High Hb	Low Hb	Med Hb	High Hb	Low Hb	Med Hb	High Hb	Low Hb	Med Hb	High Hb	Low Hb	Med Hb	High Hb	Low Hb	Med Hb	High Hb
**Cartilage loss**																		
0 (none)	55 (96.5)	49 (94.2)	51 (94.4)	54 (94.7)	49 (94.2)	50 (92.6)	46 (80.7)	45 (86.5)	41 (75.9)	56 (98.2)	50 (96.2)	50 (92.6)	77 (77.0)	66 (78.6)	62 (68.1)	27 (47.4)	23 (44.2)	18 (33.3)
1 (<10%)	1 (1.8)	3 (5.8)	2 (3.7)	2 (3.5)	2 (3.8)	3 (5.6)	8 (14.0)	3 (5.8)	11 (20.4)	0 (0.0)	2 (3.8)	3 (5.6)	14(14.0)	9 (10.7)	24 (26.4)	22 (38.6)	19 (36.5)	23 (42.6)
2 (10–75%)	1 (1.8)	0 (0.0)	1 (1.9)	1 (1.8)	1 (1.9)	1 (1.9)	3 (5.3)	3 (5.8)	2 (3.7)	1 (1.8)	0 (0.0)	1 (1.9)	9 (9.0)	8 (9.5)	4 (4.4)	8 (14.0)	8 (15.4)	11 (20.4)
3 (>75%)	0 (0.0)	0 (0.0)	0 (0.0)	0 (0.0)	0 (0.0)	0 (0.0)	0 (0)	0 (0.0)	0 (0)	0 (0.0)	0 (0.0)	0 (0.0)	0 (0.0)	1 (1.2)	1 (1.1)	0 (0.0)	2 (3.8)	2 (3.7)
**FT. cartilage loss**																		
0 (none)	56 (98.2)	52 (100.0)	54 (100)	56 (98.2)	52 (100)	54 (100.0)	55 (96.5)	51 (98.1)	53 (98.1)	56 (98.2)	52 (100.0)	52 (96.3)	94 (94.0)	80 (95.2)	85 (93.4)	53 (93.0)	44 (84.6)	49 (90.7)
1 (<10%)	0 (0.0)	0 (0.0)	0 (0.0)	1 (1.8)	0 (0.0)	0 (0.0)	1 (1.8)	0 (0.0)	0 (0.0)	0 (0.0)	0 (0.0)	2 (3.7)	3 (3.0)	2 (2.4)	4 (4.4)	4 (7.0)	4 (7.7)	5 (9.3)
2 (10–75%)	1 (1.8)	0 (0.0)	0 (0.0)	0 (0.0)	0 (0.0)	0 (0.0)	1 (1.8)	1 (1.9)	0 (0.0)	1 (1.8)	0 (0.0)	0 (0.0)	3 (3.0)	2 (2.4)	1 (1.1)	0 (0.0)	3 (5.8)	0 (0.0)
3 (>75%)	0 (0.0)	0 (0.0)	0 (0.0)	0 (0.0)	0 (0.0)	0 (0.0)	0 (0.0)	0 (0.0)	1 (1.9)	0 (0.0)	0 (0.0)	0 (0.0)	0 (0.0)	0 (0.0)	1 (1.1)	0 (0.0)	1 (1.9)	0 (0.0)
**Size of BML**																		
0 (none)	56 (98.2)	52 (100.0)	54 (100.0)	56 (98.2)	52 (100.0)	54 (100.0)	56 (98.2)	51 (98.1)	53 (98.1)	57 (100.0)	52 (100.0)	52 (96.3)	95 (95.0)	80 (95.2)	85 (93.4)	55 (96.5)	45 (86.5)	50 (92.6)
1 (<33%)	0 (0.0)	0 (0.0)	0 (0.0)	1 (1.8)	0 (0.0)	0 (0.0)	1 (1.8)	0 (0.0)	1 (1.9)	0 (0.0)	0 (0.0)	2 (3.7)	4 (4.0)	1 (1.2)	6 (6.6)	2 (3.5)	5 (9.6)	4 (7.4)
2 (33–66%)	1 (1.8)	0 (0.0)	0 (0.0)	0 (0.0)	0 (0.0)	0 (0.0)	0 (0.0)	0 (0.0)	0 (0.0)	0 (0.0)	0 (0.0)	0 (0.0)	1 (1.0)	2 (2.4)	0 (0.0)	0 (0.0)	2 (3.8)	0 (0.0)
3 (>66%)	0 (0.0)	0 (0.0)	0 (0.0)	0 (0.0)	0 (0.0)	0 (0.0)	0 (0.0)	1 (1.9)	0 (0.0)	0 (0.0)	0 (0.0)	0 (0.0)	0 (0.0)	1 (1.2)	0 (0.0)	0 (0.0)	0 (0.0)	0 (0.0)
**% That is BML**																		
0 (none)	56 (98.2)	52 (100.0)	54 (100.0)	56 (98.2)	52 (100.0)	54 (100.0)	56 (98.2)	51 (98.1)	53 (98.1)	57 (100.0)	52 (100.0)	52 (96.3)	95 (95.0)	80 (95.2)	85 (93.4)	55 (96.5)	45 (86.5)	51 (94.4)
1 (<33%)	0 (0.0)	0 (0.0)	0 (0.0)	0 (0.0)	0 (0.0)	0 (0.0)	0 (0.0)	0 (0.0)	0 (0.0)	0 (0.0)	0 (0.0)	0 (0.0)	0 (0.0)	0 (0.0)	0 (0.0)	0 (0.0)	0 (0.0)	0 (0.0)
2 (33–66%)	0 (0.0)	0 (0.0)	0 (0.0)	0 (0.0)	0 (0.0)	0 (0.0)	0 (0.0)	1 (1.9)	0 (0.0)	0 (0.0)	0 (0.0)	0 (0.0)	0 (0.0)	1 (1.2)	0 (0.0)	0 (0.0)	1 (1.9)	0 (0.0)
3 (>66%)	1 (1.8)	0 (0.0)	0 (0.0)	1 (1.8)	0 (0.0)	0 (0.0)	1 (1.8)	0 (0.0)	1 (1.9)	0 (0.0)	0 (0.0)	2 (3.7)	5 (5.0)	3 (3.6)	6 (6.6)	2 (3.5)	6 (11.5)	3 (5.6)
**Osteophyte**																		
0 (none)	52 (91.2)	47 (90.4)	52 (96.3)	53 (93.0)	48 (92.3)	51 (94.4)	53 (93.0)	49 (94.2)	51 (94.4)	49 (86.0)	46 (88.5)	50 (92.6)	79 (79.0)	68 (81.0)	83 (91.2)	30 (52.6)	31 (59.6)	28 (51.9)
1 (small or doubtful)	4 (7.0)	4 (7.7)	2 (3.7)	3 (5.3)	3 (5.8)	3 (5.6)	3 (5.3)	2 (3.8)	3 (5.6)	6 (10.5)	5 (9.6)	3 (5.6)	16 (16.0)	15 (17.9)	6 (6.6)	24 (42.1)	18 (34.6)	26 (48.1)
2 (medium)	0 (0.0)	1 (1.9)	0 (0.0)	1 (1.8)	1 (1.9)	0 (0.0)	0 (0.0)	1 (1.9)	0 (0.0)	1 (1.8)	1 (1.9)	1 (1.9)	3 (3.0)	1 (1.2)	2 (2.2)	2 (3.5)	3 (5.8)	0 (0.0)
3 (large)	1 (1.8)	0 (0.0)	0 (0.0)	0 (0.0)	0 (0.0)	0 (0.0)	1 (1.8)	0 (0.0)	0 (0.0)	1 (1.8)	0 (0.0)	0 (0.0)	2 (2.0)	0 (0.0)	0 (0.0)	1 (1.8)	0 (0.0)	0 (0.0)

In the patellofemoral joint, cartilage lesions were found in 52.6% of the joints in the low Hb tertile and 66.7% of the joints in the high Hb tertile (Table 2). Patellofemoral full-thickness cartilage lesions and BMLs were rare (Table 2). Similar distribution of patellofemoral osteophytes was observed for both the low and the high Hb tertile (Table 2), and for both males and females (Tables S4 and S5).

Other MRI features evaluated are presented in Table 3. Ganglion cysts were seen in 27.0% of MRIs in the low Hb tertile and 11.0% in the high Hb tertile. Popliteal cysts were slightly more common in the low Hb tertile (Table 3). Knee effusion was more common in the high Hb tertile (54.9%) compared to the low Hb tertile (42.0%) with most of the difference spanning from medium to large effusion (Table 3). Similar distributions were observed for both males and females (Tables S6 and S7).

**Table 3:** **Meniscal morphology, other parameters of interest and prevalence and severity of knee joint effusion according to Hb tertiles.** Data is presented as count (n) and percentage (%). Hb, hemoglobin; ACL, anterior cruciate ligament; PCL, posterior cruciate ligament.

Medial meniscal morphology	Anterior			Body			Posterior			Most severe of all 3 zones		
	Low Hb	Med Hb	High Hb	Low Hb	Med Hb	High Hb	Low Hb	Med Hb	High Hb	Low Hb	Med Hb	High Hb
Normal	98 (98.0)	83 (98.8)	89 (97.8)	89 (89.0)	69 (82.1)	83 (91.2)	90 (90.0)	70 (83.3)	82 (90.1)	89 (89.0)	67 (79.8)	82 (90.1)
Intrameniscal signal	1 (1.0)	0 (0)	0 (0)	5 (5.0)	9 (10.7)	4 (4.4)	3 (3.0)	11 (13.1)	5 (5.5)	4 (4.0)	11 (13.0)	5 (5.5)
Horizontal tear	0 (0)	1 (1.2)	0 (0)	4 (4.0)	3 (3.6)	3 (3.3)	5 (5.0)	2 (2.4)	3 (3.3)	4 (4.0)	3 (3.6)	2 (2.2)
Complex tear	1 (1.0)	0 (0.0)	1 (1.1)	2 (2.0)	2 (2.4)	0 (0.0)	1 (1.0)	1 (1.2)	0 (0)	2 (2.0)	2 (2.4)	1 (1.1)
Partial maceration	0 (0)	0 (0.0)	1 (1.1)	0 (0.0)	1 (1.2)	1 (1.1)	1 (1.0)	0 (0.0)	1 (1.1)	1 (1.0)	1 (1.2)	1 (1.1)

**Lateral meniscal morphology**	**Anterior**			**Body**			**Posterior**			**Most severe of all 3 zones**		

**Low Hb**	**Med Hb**	**High Hb**	**Low Hb**	**Med Hb**	**High Hb**	**Low Hb**	**Med Hb**	**High Hb**	**Low Hb**	**Med Hb**	**High Hb**
Normal	96 (96.0)	84 (100.0)	91 (100.0)	98 (98.0)	84 (100.0)	90 (98.9)	98 (98.0)	82 (97.6)	90 (98.9)	94 (94.0)	82 (97.6)	90 (98.9)
Intrameniscal signal	1 (1.0)	0 (0)	0 (0)	0 (0)	0 (0)	0 (0)	1 (1.0)	2 (2.4)	0 (0.0)	2 (2.0)	2 (2.4)	0 (0.0)
Horizontal tear	2 (2.0)	0 (0)	0 (0)	1 (1.0)	0 (0)	0 (0)	1 (1.0)	0 (0.0)	1 (1.1)	2 (2.0)	0 (0.0)	0 (0.0)
Complex tear	1 (1.0)	0 (0)	0 (0)	1 (1.0)	0 (0)	1 (1.1)	0 (0)	0 (0)	0 (0)	2 (2.0)	0 (0.0)	1 (1.1)
Partial maceration	0 (0)	0 (0)	0 (0)	0 (0)	0 (0)	0 (0)	0 (0)	0 (0)	0 (0)	0 (0.0)	0 (0.0)	0 (0.0)

**Other parameters of interest**	**Low Hb**	**Med Hb**	**High Hb**									

ACL tear	2 (2.0)	0 (0.0)	0 (0.0)									
ACL repair	1 (1.0)	0 (0.0)	1 (1.0)									
PCL tear	1 (1.0)	2 (2.4)	0 (0.0)									
PCL repair	0 (0.0)	0 (0.0)	0 (0.0)									
Patellar tendon signal	5 (5.0)	3 (3.6)	3 (3.3)									
Any ganglion cyst	27 (27.0)	20 (23.8)	10 (11.0)									
Pes anserine bursitis	2 (2.0)	0 (0.0)	0 (0.0)									
Infrapatellar bursa signal	20 (20.0)	13 (15.5)	14 (15.4)									
Prepatellar bursa signal	31 (31.0)	31 (36.9)	29 (31.9)									
Popliteal cyst	41 (41.0)	31 (36.9)	34 (37.4)									

	**None**			**Small**			**Medium**			**Large**		

**Low Hb**	**Med Hb**	**High Hb**	**Low Hb**	**Med Hb**	**High Hb**	**Low Hb**	**Med Hb**	**High Hb**	**Low Hb**	**Med Hb**	**High Hb**
Joint effusion	58 (58.0)	59 (70.2)	41 (45.1)	38 (38.0)	20 (23.8)	35 (38.5)	2 (2.0)	5 (6.0)	14 (15.4)	2 (2.0)	0 (0.0)	1 (1.1)

	**Normal**			**Mild**			**Moderate**			**Severe**		

**Low Hb**	**Med Hb**	**High Hb**	**Low Hb**	**Med Hb**	**High Hb**	**Low Hb**	**Med Hb**	**High Hb**	**Low Hb**	**Med Hb**	**High Hb**
Hoffa-synovitis	38 (38.0)	38 (45.2)	38 (41.8)	50 (50.0)	29 (34.5)	35 (38.5)	12 (12.0)	17 (20.2)	18 (19.8)	0 (0.0)	0 (0.0)	0 (0.0)

Overall, although minor differences in MRI features were observed between Hb tertiles, these were limited to mild abnormalities and advanced lesions were rare.

### 3.3 Associations of Hb levels with knee MRI findings

Unadjusted and adjusted associations between Hb levels (g/L) as continuous variable and knee MRI findings are presented in Tables 4 and S8–S9. In unadjusted models, Hb levels were not consistently associated with either cartilage loss, BMLs or osteophytes, and observed RRs were small overall (Tables S8–S9). Some evidence for a positive association between Hb levels and MRI findings was observed for cartilage loss and for a negative association between Hb levels and osteophytes (Table 4). RRs of similar magnitude were observed for both sexes (Table S8). In Model 1 (adjusted for sex), all RRs for cartilage loss were consistently greater but also CIs were wider, and Hb levels were positively associated with patellofemoral and any cartilage loss (Table 4). For osteophytes, all RRs had a larger reduction in risk in Model 1 than unadjusted, and Hb levels were negatively associated with tibial medial-, femoral lateral- and tibiofemoral osteophytes (Table 4). Most associations remained largely the same when also adjusted for BMI (Model 2, Table 4). In Model 2, BMI was positively associated with most knee MRI findings (Table S9). Of other MRI findings of interest, Hb levels were negatively associated with ganglion cysts and positively with the amount of knee joint effusion. The association with ganglion cysts strengthened after adjusting for sex and BMI but the significance for knee joint effusion association was lost (Table 4).

**Table 4:** **Relative Risks with 95% Confidence Intervals of individual Hb levels (g/L) for knee MRI findings.** Model 1 is adjusted for sex and Model 2 for sex and BMI∗ Indicates a logistic regression model and Odds Ratio (OR). Unmarked parameters were analyzed with Poisson regression and the result is given as Relative Risk Ratio (RR). BML, bone marrow lesion; FT, full thickness.

	Unadjusted	Model 1	Model 2
**Cartilage loss**			
Tibial medial	1.000 (0.997–1.003)	1.032 (0.941–1.133)	1.034 (0.943–1.134)
Tibial lateral	1.003 (1.000–1.007)	1.027 (0.981–1.076)	1.022 (0.973–1.073)
Femoral medial	1.001 (0.995–1.007)	1.005 (0.969–1.043)	1.002 (0.964–1.041)
Femoral lateral	1.003 (0.999–1.007)	1.027 (0.953–1.107)	1.021 (0.944–1.051)
Tibiofemoral	1.004 (0.997–1.010)	1.008 (0.978–1.039)	1.005 (0.974–1.036)
Patellofemoral	1.004 (0.996–1.013)	1.016 (1.000–1.032)	1.015 (0.999–1.032)
Any	1.007 (0.998–1.015)	1.014 (1.000–1.028)	1.013 (1.000–1.027)
**FT cartilage loss**			
Tibiofemoral	1.001 (0.997–1.005)	0.986 (0.923–1.054)	0.979 (0.909–1.053)
Patellofemoral	1.001 (0.996–1.005)	0.998 (0.961–1.035)	0.994 (0.957–1.033)
Any	1.002 (0.997–1.008)	1.000 (0.967–1.034)	0.996 (0.962–1.032)
**Size of BML**			
Tibiofemoral	1.001 (0.998–1.005)	0.999 (0.939–1.062)	0.990 (0.926–1.059)
Patellofemoral	1.002 (0.998–1.006)	1.017 (0.978–1.057)	1.016 (0.976–1.057)
Any	1.004 (0.999–1.009)	1.009 (0.976–1.044)	1.006 (0.971–1.042)
**% That is BML**			
Tibiofemoral	1.005 (0.998–1.013)	1.007 (0.938–1.082)	1.000 (0.925–1.080)
Patellofemoral	1.002 (0.994–1.010)	1.010 (0.965–1.058)	1.009 (0.962–1.058)
Any	1.007 (0.997–1.018)	1.009 (0.970–1.050)	1.006 (0.965–1.048)
**Osteophytes**			
Tibial medial	0.997 (0.993–1.001)	0.918 (0.855–0.985)	0.905 (0.836–0.979)
Tibial lateral	0.999 (0.996–1.003)	0.956 (0.901–1.014)	0.947 (0.887–1.010)
Femoral medial	0.998 (0.994–1.002)	0.935 (0.869–1.005)	0.921 (0.849–1.000)
Femoral lateral	0.997 (0.993–1.002)	0.946 (0.900–0.995)	0.938 (0.890–0.989)
Tibiofemoral	0.986 (0.960–1.012)	0.947 (0.908–0.987)	0.939 (0.899–0.982)
Patellofemoral	1.006 (0.999–1.013)	0.996 (0.978–1.014)	0.994 (0.976–1.012)
Any	1.005 (0.998–1.012)	0.994 (0.977–1.011)	0.992 (0.975–1.009)
**Other**			
Patellar tend. Signal∗	0.985 (0.933–1.041)	0.981 (0.903–1.065)	0.979 (0.901–1.064)
Any ganglion cyst ∗	0.971 (0.945–0.998)	0.942 (0.904–0.983)	0.942 (0.903–0.983)
Inf.pat. bursa signal∗	0.998 (0.970–1.026)	0.980 (0.939–1.023)	0.980 (0.939–1.023)
Prepat. Bursa signal∗	0.989 (0.967–1.012)	0.996 (0.963–1.030	0.994 (0.961–1.029)
Popliteal cyst∗	1.004 (0.983–1.026)	0.991 (0.959–1.024)	0.989 (0.958–1.022)
Joint effusion	1.013 (1.005–1.020)	1.019 (0.998–1.041)	1.016 (0.995–1.037)
Hoffa-synovitis	1.002 (0.992–1.012)	0.997 (0.983–1.012)	0.996 (0.982–1.011)

## 4 Discussion

We have previously shown, that lower Hb levels within the normal variation are associated with upregulation of HIF target genes and that Hb levels can be used as a surrogate marker of tissue oxygenation [2]. Based on our previous findings on higher Hb levels being associated with adverse metabolism and heavier body composition [2,6], and the relation of clinical factors such as higher BMI on knee MRI findings [8,26,27], we hypothesized that higher Hb levels might be associated with OA-related knee MRI findings and the associations might be mediated by heavier body composition. The above results suggest that, among this mostly asymptomatic 33-year-old cohort with sparse advanced knee OA MRI findings, Hb levels within the normal range of variation were not convincingly associated with OA-related structural changes in the knee, although a few subtle associations were observed.

Regarding clinical factors, subjects in the medium and high Hb tertiles had similar and greater BMI and waist circumference, and higher levels of fasting glucose, LDL cholesterol and bp than subjects in the low Hb tertile. Although the differences detected here were minor, these align with our previous results regarding Hb levels and metabolic dysfunction from several different and larger cohorts, where Hb levels were associated with heavier body composition and metabolic dysfunction, and the associations strengthened with age [2,6]. Moreover, as heavier body composition was the clinical factor associated with most knee MRI findings in this population [8], it is not uncalled for to suggest an interplay between Hb levels, body composition and OA-related findings might exist.

Cartilage loss in the tibiofemoral joint was largely absent or minimal across all Hb tertiles, whereas patellofemoral cartilage loss was slightly more frequent, particularly in subjects belonging to the high Hb tertile. Adjusted regression analyses showed a weak positive association between Hb levels and any cartilage loss. The association was mostly driven by patellofemoral cartilage loss but also all effect sizes regarding individual tibiofemoral joint compartments were consistently positive. It is to be noted that although BMI was positively associated with most knee MRI findings, the effects observed between Hb levels and cartilage loss for example were likely not mediated by BMI, which could suggest an effect independent of BMI. Overall, the findings cautiously suggest that higher Hb levels might be linked to cartilage loss. However, as full-thickness cartilage loss and BMLs were rare in this population, and as the possible effect studied is likely weak, studies on populations with well-characterized granular MRI data, more frequent advanced MRI findings and more statistical power, are warranted.

Osteophytes were mostly absent or small in all Hb tertiles, and differences between tertiles were minor. Inversely to that observed for cartilage loss, adjusted RRs between Hb levels and osteophytes were consistently negative, with the most convincing associations observed between Hb levels and osteophytes of the tibial medial and femoral lateral compartments. However, the associations were driven by difference in small or doubtful osteophytes as grade 2+ osteophytes in these compartments were very sparse (*n* = 2 in the tibial lateral and *n* = 4 in the femoral lateral compartment, respectively). These associations cautiously suggest higher Hb levels may have a protective effect on osteophyte formation. Importantly, small osteophytes are common especially in young adults [28,29] and may result from physiological bone remodeling rather than early knee OA [30]. Therefore, observed associations may not be related to clinically relevant osteophyte formation. Moreover, as with advanced cartilage loss, also advanced osteophytes were rare in this population, and as the possible effect observed is likely weak, studies on populations with well-characterized MRI data, more frequent advanced MRI findings and more statistical power, are warranted. Also, as with the effect observed for cartilage loss, the effect between Hb levels and osteophytes was not mediated by BMI.

Of other MRI findings of interest, ganglion cysts were negatively associated with Hb level, and joint effusion was consistently more common in subjects with higher Hb levels. Joint effusion and ganglion cysts may stem from inflammatory processes or previous trauma, however, as no difference in previous fractures or hs-CRP was observed between the Hb tertiles, it is unlikely these unspecific findings are explained by differences in previous traumas or inflammatory profile between the Hb tertiles. Effusion and synovitis are a frequent finding in OA, and in most cases not properly separable in non-contrast fluid sensitive imaging [24,31]. Effusion-synovitis has been found to mediate the worsening of OA in clinical imaging [32,33]. Although speculative, as joint effusion-synovitis is linked to both obesity and OA [34,35], the results could be suggestive for a link between higher Hb levels, heavier body composition, occurrence of intra-articular synovitis, and knee MRI findings.

Although the current study provides no mechanistic data, previous studies on the role of the HIF pathway in cartilage homeostasis and OA pathogenesis have reported data that aligns with our hypothesis of lower Hb levels being beneficial regarding joint health. Experimental studies have shown that HIF1α protects chondrocytes from apoptosis, supports ECM synthesis, and suppresses inflammatory signaling, suggesting systemic oxygenation status could positively influence joint health [36]. However, this evidence stems from *in vitro* and animal studies, using extreme hypoxia or HIF manipulation, rather than mild physiological hypoxia, which stems from for example lower Hb levels [2]. Studies linking systemic markers of mild tissue hypoxia, such as Hb levels, to OA, are scarce. In a study, the Hb levels of subjects with symptomatic knee OA were lower than that of other individuals [37]. However, the study did not account for sex differences in Hb levels nor did exclude anemic subjects. Moreover, diagnosis of OA was based on a questionnaire.

Although little prior evidence on the subject is available and Hb levels within the normal range were not strongly associated with OA features in this relatively young and healthy population, studies on older populations and with an OA diagnosis are warranted.

There are obviously some limitations to this study, many of which have been discussed earlier [8,26]. The current population was not primarily designed to address whether Hb levels are associated with knee MRI findings but to establish a longitudinal research program which aims to promote health and well-being of the population. The infrequency of advanced MRI findings and consequent lack of statistical power to detect supposedly weak effects may be the main cause no clear-cut results were obtained in this study. However, as Hb levels have been strongly associated with anthropometrics in prior studies [2,6,[38], [39], [40]] and anthropometrics are a decisive factor in OA development, it is highly unlikely that no association between Hb levels and OA exists. This is also supported by the lack of the Hb dose dependent increase of BMI in the study population which was seen in the non-included participants. Moreover, the potential association of Hb levels with OA parameters may only become visible at an older age. However, to what extent the potential association between Hb levels and knee MRI findings might be mediated by factors such as greater BMI is another story altogether.

Another limitation is our inability to assess the direct impact of oxygen levels on the knee joint. Hb levels have nevertheless been shown to be associated with oxygen consumption in a sub-population of the NFBC1966 cohort, supporting the assumption that Hb levels can be used as a surrogate measure of oxygen levels [2]. To provide a comprehensive overview of the data, the data were first analyzed across sex-specific Hb tertiles. This allows visualization of potential non-linear trends, sex-specific patterns and distribution differences and severity of MRI findings across the range of Hb levels without applying a predefined statistical model. Although parameters were first analyzed individually and then with multivariable regression models, some residual confounding could exist. The possibility of the results being based on chance is higher with a limited sample size.

A key strength of this study is the use of detailed granular knee MRI at an early stage of life, allowing investigation of preclinical OA changes. As to limitations in imaging, full-length standing radiographs for assessing axial alignment were unavailable for this study. As only non-enhanced MRI sequences were available, a definite distinction between synovitis and joint effusion would have been imprecise and was not attempted [31,41]. MOAKS does not evaluate the static MRI features of patellofemoral alignment, thus patellofemoral instability [42] as a potential confounder was not evaluated. Inter- or intra-reader reliability data has not yet been established for this study population, however, the repeatability of MOAKS has been well established in multiple previous publications [24,25,43].

The findings of preclinical studies that have linked cellular oxygen sensing mechanisms to bone and cartilage health do not conflict with our limited findings [13,14,17,36]. Although no clear associations were observed in this relatively young and healthy population, these findings do not exclude the role [[12], [13], [14],^36^]of systemic oxygen sensing mechanisms in OA development. To obtain an understanding of the importance of the Hb level as a risk indicator, one can translate the risk estimates into increased risks. For example, the results correspond to a 57.2% increase in the risk of for any cartilage loss for a 35 g/L increase in the Hb level – representing differences in normal variation of Hb levels – the corresponding increases based on Model 2 adjusted for sex and BMI. An identical increase in risk for join effusion was seen in the unadjusted data. These give a suggestion for the clinical significance of normal variation in Hb levels regarding the knee MRI findings.

Hb levels within the normal range were not robustly associated with knee OA–related structural changes on MRI in the studied population. These findings of exploratory nature suggest that in young adults, Hb levels may have limited influence on knee joint structural findings. However, as OA is a progressive disease, the potential role of Hb and oxygen-related mechanisms in joint degeneration may become more apparent later in life. Studies on populations with well-characterized MRI data, more frequent advanced MRI findings and more statistical power, are warranted.

## Author contributions

A.T. Writing - original draft, formal analysis, visualization, writing - review & editing.

J.T.: Conceptualization, writing - original draft, writing - review & editing, supervision.

A.K.: Conceptualization, data curation, writing - review & editing.

M.T. Nieminen: Funding acquisition, resources, writing - review & editing.

S.S.: Funding acquisition, resources, writing - review & editing.

M.T. Nevalainen: Project administration, resources, writing - review & editing.

P.K. Project administration, resources, writing - original draft, writing - review & editing, supervision.

The corresponding author (P.K.) takes responsibility for the overall integrity of the research article.

## Role of the funding source

This work was supported by the 10.13039/501100002341Research Council of Finland (Flagship of Advanced Mathematics for Sensing Imaging and Modelling grant #359186 and grant #354692), and the Sigrid Jusélius Foundation. NFBC1986 33-35y follow-up study received financial support from 10.13039/501100006196University of Oulu (Strategic funding from donations) and Oulu University Hospital (K65760).

## Conflict of interest

Given their role as an Associate Editor, Simo Saarakkala had no involvement in the peer-review of this article and had no access to information regarding its peer-review. Full responsibility for the editorial process for this article was delegated to another journal Editor. The authors declare no other conflict of interest.
