# Supplementary material for: Associations of hemoglobin levels with structural knee MRI findings at 33 years of age in a general population-based birth cohort
Source: Osteoarthr Cartil Open. 2026 Jun 12;8(3):100841. doi: 10.1016/j.ocarto.2026.100841 (PMC13312460; doi:10.1016/j.ocarto.2026.100841)

Supplementary material

Associations of hemoglobin levels with structural knee MRI findings at 33 years of age in a general population-based birth cohort

Atte Tapiola[a,b], Joona Tapio[a,b], Antti Kemppainen[c,d], Miika T. Nieminen[c,d], Simo Saarakkala[b,c,d], Mika T. Nevalainen[c,d], Peppi Koivunen[a,b*]

[a]Research Unit of ECM and Hypoxia, Faculty of Biochemistry and Molecular Medicine, University of Oulu, P.O. Box 5400, FIN-90014 Oulu, Finland

[b]Biocenter Oulu, University of Oulu, P.O. Box 5400, FIN-90014 Oulu, Finland

[c]Research Unit of Health Sciences and Technology, Faculty of Medicine, University of Oulu P.O. Box 5000, FI-90014 Oulu, Finland

[d]Medical Research Center Oulu, University of Oulu and Oulu University Hospital Oulu, Finland

Tables S1-S9

Figure S1

Table S1. Background and clinical characteristics of the study population and non-included participants. Data is presented as mean (M) and standard deviation (SD) for normally-distributed continuous variables, median (Mn) and interquartile range (IQR) for skewed continuous variables and count (n) and percentage (%) for count variables. *p*-Values for the study population and non-included participants were determined with one-way ANOVA for normally-distributed continuous variables, Mann-Whitney U for skewed continuous variables and Chi-square for categorical variables. M, mean; SD, standard deviation; OA, osteoarthritis; Hb, hemoglobin; BMI, body mass index; fP, fasted plasma; HDL, high-density lipoprotein; LDL, low-density lipoprotein; hs-CRP, high-sensitivity C-reactive protein.

	**Study population**		**Non-included participants**		
	**M (SD)/Mn(IQR) or n (valid %)**	**Valid n**	**M (SD)/Mn(IQR) or n (valid %)**	**Valid n**	***p*-Value**
Males n (valid %)	112 (40.7)	275	573 (40.1)	1430	0.838
Females n (valid %)	163 (59.3)	275	857 (59.9)	1430	0.838
Prior lower limb fracture n (valid %)	33 (12.0)	275	138 (11.5)	1195	0.587
Family history of knee OA n (valid %)	92 (33.5)	275	354 (30.0)	1179	0.182
Never smoker n (valid %)	103 (37.5)	275	416 (32.1)	1297	0.133
Physical activity score	14.9 (3.3)	275	15.3 (3.5)	1206	0.053
Hb (g/L)	137.3 (11.4)	275	137.9 (11.3)	1430	0.585
BMI (kg/m^2)	25.8 (4.6)	275	26.1 (4.9)	1406	0.497
Waist circumference (cm)	87.5 (12.2)	275	88.2 (13.0)	1415	0.641
Systolic blood pressure (mmHg)	112.3 (12.2)	275	112.5 (12.0)	1428	0.142
Diastolic blood pressure (mmHg)	74.5 (8.9)	275	74.8 (9.0)	1428	0.598
fP-Glucose (mmol/L)	5.0 (0.6)	275	5.0 (0.8)	1429	0.323
fP-LDL cholesterol (mmol/L)	2.8 (0.8)	275	2.7 (0.8)	1430	0.349
hs-CRP (mg/L)	0.7 (0.3-1.6)	275	0.7 (0.4-1.5)	1430	0.987

Table S2. Background and clinical characteristics of the non-included participants in Hb tertiles. Data is presented as mean (M) and standard deviation (SD) for normally-distributed continuous variables, median (Mn) and interquartile range (IQR) for skewed continuous variables and count (n) and percentage (%) for count variables. M, mean; SD, standard deviation; OA, osteoarthritis; Hb, hemoglobin; BMI, body mass index; fP, fasted plasma; HDL, high-density lipoprotein; LDL, low-density lipoprotein; hs-CRP, high-sensitivity C-reactive protein.

	**Low Hb**<br>**M (SD)/Mn(IQR) or n (valid %)**	**Medium Hb**<br>**M (SD)/Mn(IQR) or n (valid %)**	**High Hb**<br>**M (SD)/Mn(IQR) or n (valid %)**
**All participants**			
Males n (valid %), valid n = 1430	217 (43.5)	170 (34.6)	186 (42.3)
Females n (valid %), valid n = 1430	282 (56.5)	321 (65.4)	254 (57.7)
Prior lower limb fracture n (valid %), valid n = 1195	44 (10.5)	55 (13.2)	39 (10.9)
Family history of knee OA n (valid %), valid n = 1179	123 (29.7)	125 (30.6)	106 (29.7)
Never smoker n (valid %), valid n = 1297	147 (32.8)	143 (32.1)	126 (31.2)
Physical activity score, valid n = 1206	15.5 (3.3)	15.2 (3.5)	15.1 (3.5)
Hb (g/L), valid n = 1430	130.8 (9.4)	137.0 (8.5)	146.9 (9.6)
BMI (kg/m^2), valid n =1406	25.0 (4.1)	26.0 (4.9)	27.5 (5.5)
Waist circumference (cm), valid n = 1415	85.8 (12.0)	87.5 (12.8)	91.7 (13.5)
Systolic blood pressure (mmHg), valid n = 1428	112.2 (12.9)	111.3 (12.9)	114.3 (13.1)
Diastolic blood pressure (mmHg), valid n = 1428	73.3 (8.7)	74.4 (8.8)	76.9 (9.2)
fP-Glucose (mmol/L), valid n = 1429	5.0 (0.6)	5.0 (0.7)	5.2 (1.0)
fP-LDL cholesterol (mmol/L), valid n =1430	2.6 (0.7)	2.7 (0.8)	2.9 (0.8)
hs-CRP (mg/L), valid n = 1430	0.6 (0.3-1.4)	0.7 (0.4-1.5)	0.8 (0.4-1.8)

Table S3. Magnetic resonance imaging sequence parameters. Similar MRI protocol was used for all subjects with identical repetition time (TR), echo time (TE) and other imaging parameters in different pulse sequences, with sagittal slice orientation and a slice gap of 20 %. All sequences were isotropic (i.e., 3D). DESS; Double Echo Steady State, FA; flip angle, FOV; field-of-view, fs; fat saturation, PD; proton density, sag; sagittal, SPACE; Sampling Perfection with Application-optimized Contrasts using different flip angle Evolutions.

	TR	TE	Slice thickness	Spacing	FA	Matrix size	FOV	Resolution
T2 SPACE fs sag	1000 ms	123 ms	0.6 mm	0.6 mm	120	256 x 256	16 x 16 cm^2	0.6 x 0.6 mm
PD SPACE sag	900 ms	76 ms	0.6 mm	0.6 mm	120	256 x 256	16 x 16 cm^2	0.6 x 0.6 mm
3D DESS sag	14.1 ms	5 ms	0.6 mm	0.6 mm	25	256 x 256	16 x 16 cm^2	0.59 x 0.59 mm

Table S4. Counts of the most severe in-patient MRI-detected cartilage lesions, BMLs and osteophytes in the tibiofemoral and patellofemoral joint regions according to Hb tertiles in males. Data is presented as count (n) and percentage (%). Hb, hemoglobin; FT, full thickness; BML; bone marrow lesion.

	Tibial medial			Tibial lateral			Femoral medial			Femoral lateral			Tibiofemoral			Patellofemoral		
**Hb**	**Low Hb**	**Med Hb**	**High Hb**	**Low Hb**	**Med Hb**	**High Hb**	**Low Hb**	**Med Hb**	**High Hb**	**Low Hb**	**Med Hb**	**High Hb**	**Low Hb**	**Med Hb**	**High Hb**	**Low Hb**	**Med Hb**	**High Hb**
**Cartilage loss**																		
0 (none)	41 (95.3)	32 (100.0)	34 (91.9)	39 (90.7)	27 (84.4)	31 (83.8)	37 (86.0)	26 (81.3)	28 (75.7)	40 (93.0)	29 (90.6)	33 (89.2)	33 (76.7)	23 (71.9)	26 (70.3)	20 (46.5)	19 (59.4)	15 (40.5)
1 (< 10%)	2 (4.7)	0 (0.0)	3 (8.1)	4 (9.3)	4 (12.5)	6 (16.2)	1 (2.3)	3 (9.4)	7 (18.9)	2 (4.7)	2 (6.3)	3 (8.1)	4 (9.3)	4 (12.5)	9 (24.3)	15 (34.9)	5 (15.6)	15 (40.5)
2 (10 - 75%)	0 (0.0)	0 (0.0)	0 (0.0)	0 (0.0)	1 (3.1)	0 (0.0)	5 (11.6)	3 (9.4)	2 (5.4)	1 (2.3)	1 (3.1)	0 (0.0)	6 (14.0)	5 (15.6)	1 (2.7)	7 (16.3)	8 (25.0)	4 (10.8)
3 (> 75%)	0 (0.0)	0 (0.0)	0 (0.0)	0 (0.0)	0 (0.0)	0 (0.0)	0 (0)	0 (0.0)	0 (0)	0 (0.0)	0 (0.0)	1 (2.7)	0 (0.0)	0 (0.0)	1 (2.7)	1 (2.3)	0 (0.0)	3 (8.1)
**FT. cartilage loss**																		
0 (none)	43 (100.0)	32 (100.0)	36 (97.3)	43 (100.0)	31 (96.9)	34 (91.9)	42 (97.7)	30 (93.8)	36 (97.3)	41 (95.3)	31 (96.9)	36 (97.3)	40 (93.0)	29 (90.6)	34 (91.9)	37 (86.0)	27 (84.4)	33 (89.2)
1 (< 10%)	0 (0.0)	0 (0.0)	1 (2.7)	0 (0.0)	1 (3.1)	3 (8.1)	0 (0.0)	1 (3.1)	1 (2.7)	1 (2.3)	1 (3.1)	0 (0.0)	1 (2.3)	2 (6.3)	2 (5.4)	5 (11.6)	4 (12.5)	3 (8.1)
2 (10 - 75%)	0 (0.0)	0 (0.0)	0 (0.0)	0 (0.0)	0 (0.0)	0 (0.0)	1 (2.3)	1 (3.1)	0 (0.0)	1 (2.3)	0 (0.0)	1 (2.7)	2 (4.7)	1 (3.1)	1 (2.7)	1 (2.3)	1 (3.1)	1 (2.7)
3 (> 75%)	0 (0.0)	0 (0.0)	0 (0.0)	0 (0.0)	0 (0.0)	0 (0.0)	0 (0.0)	0 (0.0)	0 (0.0)	0 (0.0)	0 (0.0)	0 (0.0)	0 (0.0)	0 (0.0)	0 (0.0)	0 (0.0)	0 (0.0)	0 (0.0)
**Size of BML**																		
0 (none)	43 (100.0)	32 (100.0)	36 (97.3)	42 (97.7)	31 (96.9)	34 (91.9)	42 (97.7)	31 (96.9)	36 (96.9)	42 (97.7)	31 (96.9)	36 (96.9)	40 (93.0)	29 (90.6)	34 (91.9)	39 (90.7)	28 (87.5)	34 (91.9)
1 (< 33%)	0 (0.0)	0 (0.0)	1 (2.7)	1 (2.3)	1 (3.1)	3 (8.1)	1 (2.3)	1 (3.1)	1 (2.7)	1 (2.3)	0 (0.0)	1 (2.7)	3 (7.0)	1 (3.1)	3 (8.1)	3 (7.0)	3 (9.4)	2 (5.4)
2 (33 - 66%)	0 (0.0)	0 (0.0)	0 (0.0)	0 (0.0)	0 (0.0)	0 (0.0)	0 (0.0)	0 (0.0)	0 (0.0)	0 (0.0)	1 (3.1)	0 (0.0)	0 (0.0)	2 (6.3)	0 (0.0)	0 (0.0)	1 (3.1)	1 (2.7)
3 (> 66%)	0 (0.0)	0 (0.0)	0 (0.0)	0 (0.0)	0 (0.0)	0 (0.0)	0 (0.0)	0 (0.0)	0 (0.0)	0 (0.0)	0 (0.0)	0 (0.0)	0 (0.0)	0 (0.0)	0 (0.0)	1 (2.3)	0 (0.0)	0 (0.0)
**% that is BML**																		
0 (none)	43 (100.0)	32 (100.0)	36 (97.3)	42 (97.7)	31 (96.9)	34 (91.0)	42 (97.7)	31 (96.9)	36 (97.3)	42 (97.7)	31 (96.9)	36 (97.3)	40 (93.0)	29 (90.6)	34 (91.9)	40 (93.0)	28 (87.5)	35 (94.6)
1 (< 33%)	0 (0.0)	0 (0.0)	0 (0.0)	0 (0.0)	0 (0.0)	0 (0.0)	0 (0.0)	0 (0.0)	0 (0.0)	0 (0.0)	0 (0.0)	0 (0.0)	0 (0.0)	0 (0.0)	0 (0.0)	0 (0.0)	0 (0.0)	0 (0.0)
2 (33 - 66%)	0 (0.0)	0 (0.0)	0 (0.0)	0 (0.0)	0 (0.0)	0 (0.0)	0 (0.0)	0 (0.0)	0 (0.0)	0 (0.0)	0 (0.0)	0 (0.0)	0 (0.0)	0 (0.0)	0 (0.0)	0 (0.0)	0 (0.0)	0 (0.0)
3 (> 66%)	0 (0.0)	0 (0.0)	1 (2.7)	1 (2.3)	1 (3.1)	3 (8.1)	1 (2.3)	1 (3.1)	1 (2.7)	1 (2.3)	1 (3.1)	1 (2.7)	3 (7.0)	3 (9.4)	3 (8.1)	3 (7.0)	4 (12.5)	2 (5.4)
**Osteophyte**																		
0 (none)	37 (86.0)	32 (100.0)	36 (97.3)	38 (88.4)	29 (90.6)	36 (97.3)	38 (88.4)	30 (93.8)	36 (97.3)	33 (76.7)	29 (90.6)	34 (91.9)	32 (74.4)	25 (78.1)	34 (91.9)	18 (41.9)	12 (37.5)	16 (43.2)
1 (small or doubtful)	4 (9.3)	0 (0.0)	0 (0.0)	4 (9.3)	3 (9.4)	0 (0.0)	3 (7.0)	2 (6.3)	0 (0.0)	9 (20.9)	3 (9.4)	3 (8.1)	8 (18.6)	7 (21.9)	2 (5.4)	20 (46.5)	18 (56.3)	16 (43.2)
2 (medium)	1 (2.3)	0 (0.0)	1 (2.7)	0 (0.0)	0 (0.0)	1 (2.7)	1 (2.3)	0 (0.0)	1 (2.7)	0 (0.0)	0 (0.0)	0 (0.0)	2 (4.7)	0 (0.0)	1 (2.7)	4 (9.3)	2 (6.3)	5 (13.5)
3 (large)	1 (2.3)	0 (0.0)	0 (0.0)	1 (2.3)	0 (0.0)	0 (0.0)	1 (2.3)	0 (0.0)	0 (0.0)	1 (2.3)	0 (0.0)	0 (0.0)	1 (2.3)	0 (0.0)	0 (0.0)	1 (2.3)	0 (0.0)	0 (0.0)

Table S5. Counts of the most severe in-patient MRI-detected cartilage lesions, BMLs and osteophytes in the tibiofemoral and patellofemoral joint regions according to Hb tertiles in females. Data is presented as count (n) and percentage (%). Hb, hemoglobin; FT, full thickness; BML; bone marrow lesion.

	Tibial medial			Tibial lateral			Femoral medial			Femoral lateral			Tibiofemoral			Patellofemoral		
**Hb**	**Low Hb**	**Med Hb**	**High Hb**	**Low Hb**	**Med Hb**	**High Hb**	**Low Hb**	**Med Hb**	**High Hb**	**Low Hb**	**Med Hb**	**High Hb**	**Low Hb**	**Med Hb**	**High Hb**	**Low Hb**	**Med Hb**	**High Hb**
**Cartilage loss**																		
0 (none)	55 (96.5)	49 (94.2)	51 (94.4)	54 (94.7)	49 (94.2)	50 (92.6)	46 (80.7)	45 (86.5)	41 (75.9)	56 (98.2)	50 (96.2)	50 (92.6)	44 (77.2)	43 (82.7)	36 (66.7)	27 (47.4)	23 (44.2)	18 (33.3)
1 (< 10%)	1 (1.8)	3 (5.8)	2 (3.7)	2 (3.5)	2 (3.8)	3 (5.6)	8 (14.0)	3 (5.8)	11 (20.4)	0 (0.0)	2 (3.8)	3 (5.6)	10 (17.5)	5 (9.6)	15 (27.8)	22 (38.6)	19 (36.5)	23 (42.6)
2 (10 - 75%)	1 (1.8)	0 (0.0)	1 (1.9)	1 (1.8)	1 (1.9)	1 (1.9)	3 (5.3)	3 (5.8)	2 (3.7)	1 (1.8)	0 (0.0)	1 (1.9)	3 (5.3)	3 (5.8)	3 (5.6)	8 (14.0)	8 (15.4)	11 (20.4)
3 (> 75%)	0 (0.0)	0 (0.0)	0 (0.0)	0 (0.0)	0 (0.0)	0 (0.0)	0 (0)	0 (0.0)	0 (0)	0 (0.0)	0 (0.0)	0 (0.0)	0 (0.0)	1 (1.9)	0 (0.0)	0 (0.0)	2 (3.8)	2 (3.7)
**FT. cartilage loss**																		
0 (none)	56 (98.2)	52 (100.0)	54 (100.0)	56 (98.2)	52 (100.0)	54 (100.0)	55 (96.5)	51 (98.1)	53 (98.1)	56 (98.2)	52 (100.0)	52 (96.3)	54 (94.7)	51 (98.1)	51 (94.4)	53 (93.0)	44 (84.6)	49 (90.7)
1 (< 10%)	0 (0.0)	0 (0.0)	0 (0.0)	1 (1.8)	0 (0.0)	0 (0.0)	1 (1.8)	0 (0.0)	0 (0.0)	0 (0.0)	0 (0.0)	2 (3.7)	2 (3.5)	0 (0.0)	2 (3.7)	4 (7.0)	4 (7.7)	5 (9.3)
2 (10 - 75%)	1 (1.8)	0 (0.0)	0 (0.0)	0 (0.0)	0 (0.0)	0 (0.0)	1 (1.8)	1 (1.9)	0 (0.0)	1 (1.8)	0 (0.0)	0 (0.0)	1 (1.8)	1 (1.9)	0 (0.0)	0 (0.0)	3 (5.8)	0 (0.0)
3 (> 75%)	0 (0.0)	0 (0.0)	0 (0.0)	0 (0.0)	0 (0.0)	0 (0.0)	0 (0.0)	0 (0.0)	1 (1.9)	0 (0.0)	0 (0.0)	0 (0.0)	0 (0.0)	0 (0.0)	1 (1.9)	0 (0.0)	1 (1.9)	0 (0.0)
**Size of BML**																		
0 (none)	56 (98.2)	52 (100.0)	54 (100.0)	56 (98.2)	52 (100.0)	54 (100.0)	56 (98.2)	51 (98.1)	53 (98.1)	57 (100.0)	52 (100.0)	52 (96.3)	55 (96.5)	51 (98.1)	51 (94.4)	55 (96.5)	45 (86.5)	50 (92.6)
1 (< 33%)	0 (0.0)	0 (0.0)	0 (0.0)	1 (1.8)	0 (0.0)	0 (0.0)	1 (1.8)	0 (0.0)	1 (1.9)	0 (0.0)	0 (0.0)	2 (3.7)	1 (1.8)	0 (0.0)	3 (5.6)	2 (3.5)	5 (9.6)	4 (7.4)
2 (33 - 66%)	1 (1.8)	0 (0.0)	0 (0.0)	0 (0.0)	0 (0.0)	0 (0.0)	0 (0.0)	0 (0.0)	0 (0.0)	0 (0.0)	0 (0.0)	0 (0.0)	1 (1.8)	0 (0.0)	0 (0.0)	0 (0.0)	2 (3.8)	0 (0.0)
3 (> 66%)	0 (0.0)	0 (0.0)	0 (0.0)	0 (0.0)	0 (0.0)	0 (0.0)	0 (0.0)	1 (1.9)	0 (0.0)	0 (0.0)	0 (0.0)	0 (0.0)	0 (0.0)	1 (1.9)	0 (0.0)	0 (0.0)	0 (0.0)	0 (0.0)
**% that is BML**																		
0 (none)	56 (98.2)	52 (100.0)	54 (100.0)	56 (98.2)	52 (100.0)	54 (100.0)	56 (98.2)	51 (98.1)	53 (98.1)	57 (100.0)	52 (100.0)	52 (96.3)	55 (96.5)	51 (98.1)	51 (94.4)	55 (96.5)	45 (86.5)	51 (94.4)
1 (< 33%)	0 (0.0)	0 (0.0)	0 (0.0)	0 (0.0)	0 (0.0)	0 (0.0)	0 (0.0)	0 (0.0)	0 (0.0)	0 (0.0)	0 (0.0)	0 (0.0)	0 (0.0)	0 (0.0)	0 (0.0)	0 (0.0)	0 (0.0)	0 (0.0)
2 (33 - 66%)	0 (0.0)	0 (0.0)	0 (0.0)	0 (0.0)	0 (0.0)	0 (0.0)	0 (0.0)	1 (1.9)	0 (0.0)	0 (0.0)	0 (0.0)	0 (0.0)	0 (0.0)	1 (1.9)	0 (0.0)	0 (0.0)	1 (1.9)	0 (0.0)
3 (> 66%)	1 (1.8)	0 (0.0)	0 (0.0)	1 (1.8)	0 (0.0)	0 (0.0)	1 (1.8)	0 (0.0)	1 (1.9)	0 (0.0)	0 (0.0)	2 (3.7)	2 (3.5)	0 (0.0)	3 (5.6)	2 (3.5)	6 (11.5)	3 (5.6)
**Osteophyte**																		
0 (none)	52 (91.2)	47 (90.4)	52 (96.3)	53 (93.0)	48 (92.3)	51 (94.4)	53 (93.0)	49 (94.2)	51 (94.4)	49 (86.0)	46 (88.5)	50 (92.6)	47 (82.5)	43 (82.7)	49 (90.7)	30 (52.6)	31 (59.6)	28 (51.9)
1 (small or doubtful)	4 (7.0)	4 (7.7)	2 (3.7)	3 (5.3)	3 (5.8)	3 (5.6)	3 (5.3)	2 (3.8)	3 (5.6)	6 (10.5)	5 (9.6)	3 (5.6)	8 (14.0)	8 (15.4)	4 (7.4)	24 (42.1)	18 (34.6)	26 (48.1)
2 (medium)	0 (0.0)	1 (1.9)	0 (0.0)	1 (1.8)	1 (1.9)	0 (0.0)	0 (0.0)	1 (1.9)	0 (0.0)	1 (1.8)	1 (1.9)	1 (1.9)	1 (1.8)	1 (1.9)	1 (1.9)	2 (3.5)	3 (5.8)	0 (0.0)
3 (large)	1 (1.8)	0 (0.0)	0 (0.0)	0 (0.0)	0 (0.0)	0 (0.0)	1 (1.8)	0 (0.0)	0 (0.0)	1 (1.8)	0 (0.0)	0 (0.0)	1 (1.8)	0 (0.0)	0 (0.0)	1 (1.8)	0 (0.0)	0 (0.0)

Table S6. Meniscal morphology, other parameters of interest and prevalence and severity of knee joint effusion according to Hb tertiles in males. Data is presented as count (n) and percentage (%). Hb, hemoglobin; ACL, anterior cruciate ligament; PCL, posterior cruciate ligament.

**Medial meniscal morphology**	**Anterior**			**Body**			**Posterior**			**Most severe of all 3 zones**		
	**Low Hb**	**Med Hb**	**High Hb**	**Low Hb**	**Med Hb**	**High Hb**	**Low Hb**	**Med Hb**	**High Hb**	**Low Hb**	**Med Hb**	**High Hb**
Normal	41 (95.3)	32 (100.0)	35 (94.6)	37 (86.0)	24 (75.0)	33 (89.2)	38 (88.4)	24 (75.0)	33 (89.2)	37 (86.0)	23 (71.9)	33 (89.2)
Intrameniscal signal	1 (2.3)	0 (0)	0 (0)	2 (4.7)	5 (15.6)	2 (5.4)	1 (2.3)	6 (18.8)	2 (5.4)	2 (4.7)	6 (18.8)	2 (5.4)
Horizontal tear	0 (0)	0 (0)	0 (0)	2 (4.7)	2 (6.3)	1 (2.7)	3 (7.0)	1 (3.1)	1 (2.7)	2 (4.7)	5 (9.6)	0 (0.0)
Complex tear	1 (2.3)	0 (0.0)	1 (2.7)	2 (4.7)	1 (3.1)	0 (0.0)	1 (2.3)	1 (3.1)	0 (0)	2 (4.7)	1 (3.1)	1 (2.7)
Partial maceration	0 (0)	0 (0.0)	1 (2.7)	0 (0.0)	0 (0.0)	1 (2.7)	0 (0.0)	0 (0.0)	1 (2.7)	0 (0.0)	0 (0.0)	1 (2.7)
**Lateral meniscal morphology**	**Anterior**			**Body**			**Posterior**			**Most severe of all 3 zones**		
	**Low Hb**	**Med Hb**	**High Hb**	**Low Hb**	**Med Hb**	**High Hb**	**Low Hb**	**Med Hb**	**High Hb**	**Low Hb**	**Med Hb**	**High Hb**
Normal	42 (97.7)	32 (100.0)	37 (100.0)	42 (97.7)	32 (100.0)	36 (97.3)	41 (95.3)	31 (96.9)	36 (97.3)	40 (93.0)	31 (96.9)	36 (97.3)
Intrameniscal signal	1 (2.3)	0 (0)	0 (0)	0 (0)	0 (0)	0 (0)	1 (2.3)	1 (3.1)	0 (0.0)	2 (4.7)	1 (3.1)	0 (0.0)
Horizontal tear	0 (0)	0 (0)	0 (0)	0 (0)	0 (0)	0 (0)	1 (2.3)	0 (0.0)	1 (2.7)	0 (0.0)	0 (0.0)	0 (0.0)
Complex tear	0 (0)	0 (0)	0 (0)	1 (2.3)	0 (0)	1 (2.7)	0 (0)	0 (0)	0 (0)	1 (2.3)	0 (0.0)	1 (2.7)
Partial maceration	0 (0)	0 (0)	0 (0)	0 (0)	0 (0)	0 (0)	0 (0)	0 (0)	0 (0)	0 (0.0)	0 (0.0)	0 (0.0)
**Other parameters of interest**	**Low Hb**	**Med Hb**	**High Hb**									
ACL tear	1 (2.3)	0 (0.0)	0 (0.0)									
ACL repair	1 (2.3)	0 (0.0)	1 (2.7)									
PCL tear	0 (0.0)	1 (3.1)	0 (0.0)									
PCL repair	0 (0.0)	0 (0.0)	0 (0.0)									
Patellar tendon signal	2 (4.7)	0 (0.0)	2 (5.4)									
Any ganglion cyst	12 (27.9)	5 (15.6)	5 (13.6)									
Pes anserine bursitis	0 (0.0)	0 (0.0)	0 (0.0)									
Infrapatellar bursa signal	10 (23.3)	5 (15.6)	6 (16.2)									
Prepatellar bursa signal	14 (32.6)	10 (31.3)	9 (24.3)									
Popliteal cyst	16 (37.2)	13 (40.6)	18 (48.6)									
	**None**			**Small**			**Medium**			**Large**		
	**Low Hb**	**Med Hb**	**High Hb**	**Low Hb**	**Med Hb**	**High Hb**	**Low Hb**	**Med Hb**	**High Hb**	**Low Hb**	**Med Hb**	**High Hb**
Joint effusion	20 (46.5)	20 (62.5)	14 (37.8)	20 (46.5)	8 (25.0)	16 (43.2)	1 (2.3)	4 (12.5)	6 (16.2)	2 (4.7)	0 (0.0)	1 (2.7)
	**Normal**			**Mild**			**Moderate**			**Severe**		
Hb	**Low Hb**	**Med Hb**	**High Hb**	**Low Hb**	**Med Hb**	**High Hb**	**Low Hb**	**Med Hb**	**High Hb**	**Low Hb**	**Med Hb**	**High Hb**
Hoffa-synovitis	14 (32.6)	12 (37.5)	16 (43.2)	22 (51.2)	16 (50.0)	12 (32.4)	7 (16.3)	4 (12.5)	9 (24.3)	0 (0.0)	0 (0.0)	0 (0.0)

Table S7. Meniscal morphology, other parameters of interest and prevalence and severity of knee joint effusion according to Hb tertiles in females. Data is presented as count (n) and percentage (%). Hb, hemoglobin; ACL, anterior cruciate ligament; PCL, posterior cruciate ligament.

**Medial meniscal morphology**	**Anterior**			**Body**			**Posterior**			**Most severe of all 3 zones**		
Hb	**Low Hb**	**Med Hb**	**High Hb**	**Low Hb**	**Med Hb**	**High Hb**	**Low Hb**	**Med Hb**	**High Hb**	**Low Hb**	**Med Hb**	**High Hb**
Normal	57 (100.0)	51 (98.1)	54 (100.0)	52 (91.2)	45 (86.5)	50 (92.6)	52 (91.2)	46 (88.5)	49 (90.7)	52 (91.2)	44 (84.6)	33 (89.2)
Intrameniscal signal	0 (0)	0 (0)	0 (0)	3 (5.3)	4 (7.7)	2 (3.7)	2 (3.5)	5 (9.6)	3 (5.6)	2 (3.5)	5 (9.6)	3 (5.6)
Horizontal tear	0 (0)	1 (1.9)	0 (0)	2 (3.5)	1 (1.9)	2 (3.7)	2 (3.5)	1 (1.9)	2 (3.7)	2 (3.5)	1 (1.9)	2 (3.7)
Complex tear	0 (0)	0 (0.0)	0 (0)	0 (0.0)	1 (1.9)	0 (0.0)	0 (0.0)	0 (0.0)	0 (0)	0 (0.0)	1 (1.9)	0 (0.0)
Partial maceration	0 (0)	0 (0.0)	0 (0)	0 (0.0)	1 (1.9)	0 (0.0)	1 (1.8)	0 (0.0)	0 (0)	1 (1.8)	1 (1.9)	0 (0.0)
**Lateral meniscal morphology**	**Anterior**			**Body**			**Posterior**			**Most severe of all 3 zones**		
Hb	**Low Hb**	**Med Hb**	**High Hb**	**Low Hb**	**Med Hb**	**High Hb**	**Low Hb**	**Med Hb**	**High Hb**	**Low Hb**	**Med Hb**	**High Hb**
Normal	54 (94.7)	52 (100.0)	54 (100.0)	56 (98.2)	52 (100.0)	54 (100.0)	57 (100.0)	51 (98.1)	36 (97.3)	54 (94.7)	51 (98.1)	54 (100.0)
Intrameniscal signal	0 (0.0)	0 (0)	0 (0)	0 (0)	0 (0)	0 (0)	0 (0)	1 (1.9)	0 (0.0)	0 (0.0)	1 (1.9)	0 (0.0)
Horizontal tear	2 (3.5)	0 (0)	0 (0)	1 (1.8)	0 (0)	0 (0)	0 (0)	0 (0.0)	1 (2.7)	2 (3.5)	0 (0.0)	0 (0.0)
Complex tear	1 (1.8)	0 (0)	0 (0)	0 (0)	0 (0)	0 (0)	0 (0)	0 (0)	0 (0)	1 (1.8)	0 (0.0)	0 (0.0)
Partial maceration	0 (0)	0 (0)	0 (0)	0 (0)	0 (0)	0 (0)	0 (0)	0 (0)	0 (0)	0 (0.0)	0 (0.0)	0 (0.0)
**Other parameters of interest**	**Low Hb**	**Med Hb**	**High Hb**									
ACL tear	1 (1.8)	0 (0.0)	0 (0.0)									
ACL repair	0 (0.0)	0 (0.0)	0 (0.0)									
PCL tear	1 (1.8)	1 (1.9)	0 (0.0)									
PCL repair	0 (0.0)	0 (0.0)	0 (0.0)									
Patellar tendon signal	3 (5.3)	3 (5.8)	1 (1.9)									
Any ganglion cyst	15 (26.3)	15 (28.8)	5 (9.3)									
Pes anserine bursitis	2 (3.5)	0 (0.0)	0 (0.0)									
Infrapatellar bursa signal	10 (17.5)	8 (15.4)	8 (14.8)									
Prepatellar bursa signal	17 (29.8)	21 (40.4)	20 (37.0)									
Popliteal cyst	25 (43.9)	18 (34.6)	16 (29.6)									
	**None**			**Small**			**Medium**			**Large**		
Hb	**Low Hb**	**Med Hb**	**High Hb**	**Low Hb**	**Med Hb**	**High Hb**	**Low Hb**	**Med Hb**	**High Hb**	**Low Hb**	**Med Hb**	**High Hb**
Joint effusion	38 (66.7)	39 (75.0)	27 (50.0)	18 (31.6)	12 (23.1)	19 (35.2)	1 (1.8)	1 (1.9)	8 (14.8)	0 (0.0)	0 (0.0)	0 (0.0)
	**Normal**			**Mild**			**Moderate**			**Severe**		
Hb	**Low Hb**	**Med Hb**	**High Hb**	**Low Hb**	**Med Hb**	**High Hb**	**Low Hb**	**Med Hb**	**High Hb**	**Low Hb**	**Med Hb**	**High Hb**
Hoffa-synovitis	24 (42.1)	26 (50.0)	22 (40.7)	28 (49.1)	13 (25.0)	23 (42.6)	5 (8.8)	13 (25.0)	9 (16.7)	0 (0.0)	0 (0.0)	0 (0.0)

Table S8. Unadjusted Relative Risks or Odds Ratios with 95% Confidence Intervals of individual Hb levels (g/L) for knee MRI findings in males and females. *Indicates a logistic regression model and odds ratio (OR). Unmarked parameters were analyzed with Poisson regression and the result is given as relative risk ratio (RR). BML, bone marrow lesion; FT, full thickness.

	**Males**	**Females**
**Cartilage loss**		
Tibial medial	1.000 (0.995-1.005)	1.003 (0.994-1.012)
Tibial lateral	1.002 (0.995-1.009)	1.004 (0.996-1.011)
Femoral medial	1.001 (0.985-1.017)	1.002 (0.990-1.014)
Femoral lateral	1.000 (0.990-1.010)	1.004 (0.996-1.013)
Tibiofemoral	0.996 (0.980-1.013)	1.008 (0.995-1.022)
Patellofemoral	1.006 (0.987-1.025)	1.018 (1.001-1.035)
Any	1.005 (0.986-1.024)	1.019 (1.002-1.036)
**FT cartilage loss**		
Tibiofemoral	0.998 (0.989-1.007)	0.999 (0.992-1.007)
Patellofemoral	1.000 (0.992-1.009)	0.999 (0.992-1.006)
Any	1.000 (0.989-1.011)	1.000 (0.991-1.010)
**Size of BML**		
Tibiofemoral	1.000 (0.993-1.007)	1.000 (0.994-1.006)
Patellofemoral	1.003 (0.996-1.010)	1.001 (0.995-1.007)
Any	1.003 (0.993-1.013)	1.001 (0.993-1.009)
**% that is BML**		
Tibiofemoral	1.000 (0.978-1.022)	1.002 (0.990-1.015)
Patellofemoral	1.003 (0.991-1.016)	1.002 (0.985-1.018)

Any	1.003 (0.979-1.028)	1.004 (0.984-1.024)
**Osteophytes**		
Tibial medial	0.991 (0.981-1.001)	0.994 (0.985-1.004)
Tibial lateral	0.993 (0.986-1.001)	0.998 (0.991-1.006)
Femoral medial	0.991 (0.981-1.002)	0.996 (0.987-1.006)
Femoral lateral	0.990 (0.981-0.999)	0.994 (0.982-1.005)
Tibiofemoral	0.987 (0.974-0.999)	0.992 (0.980-1.003)
Patellofemoral	1.002 (0.985-1.019)	0.994 (0.981-1.007)
Any	0.997 (0.981-1.014)	0.995 (0.982-1.008)
**Other**		
Patellar tend. signal*	0.994 (0.876-1.129)	0.971 (0.870-1.084)
Any ganglion cyst *	0.935 (0.878-0.997)	0.948 (0.896-1.003)
Inf.pat. bursa signal*	0.972 (0.914-1.033)	0.988 (0.932-1.048)
Prepat. bursa signal*	0.962 (0.913-1.015)	1.021 (0.977-1.068)
Popliteal cyst*	1.025 (0.976-1.075)	0.962 (0.918-1.007)
Joint effusion	1.006 (0.987-1.025)	1.014 (1.000-1.028)
Hoffa-synovitis	0.991 (0.970-1.011)	1.003 (0.983-1.024)

Table S9. Relative Risks or Odds Ratios with 95% Confidence Intervals from multivariable regression model for knee MRI findings. *Indicates a logistic regression model and odds ratio (OR). Unmarked parameters were analyzed with Poisson regression and the result is given as relative risk ratio (RR). Hb, hemoglobin; BMI, body mass index; BML, bone marrow lesion; FT, full thickness.

	**Model 1**		**Model 2**		
	**Hb (g/L)**	**Male sex**	**Hb (g/L)**	**Male sex**	**BMI**
**Cartilage loss**					
Tibial medial	1.032 (0.941-1.133)	0.418 (0.054-3.245)	1.034 (0.943-1.134)	0.534 (0.056-5.082)	1.187 (1.109-1.207)
Tibial lateral	1.027 (0.981-1.076)	1.125 (0.335-3.786)	1.022 (0.973-1.073)	1.346 (0.383-4.732	1.107 (1.032-1.187)
Femoral medial	1.005 (0.969-1.043)	1.004 (0.415-2.426)	1.002 (0.964-1.041)	1.166 (0.455-2.990)	1.094 (1.046-1.145)
Femoral lateral	1.027 (0.953-1.107)	1.421 (0.240-8.417)	1.021 (0.944-1.051)	1.742 (0.276-10.981)	1.111 (1.016-1.214)
Tibiofemoral	1.008 (0.978-1.039)	1.094 (0.524-2.284)	1.005 (0.974-1.036)	1.225 (0.573-2.622)	1.075 (1.032-1.119)
Patellofemoral	1.016 (1.000-1.032)	0.723 (0.492-1.063)	1.015 (0.999-1.032)	0.732 (0.496-1.080)	1.012 (0.987-1.038)
Any	1.014 (1.000-1.028)	0.817 (0.585-1.140)	1.013 (1.000-1.027)	0.829 (0.591-1.161)	1.014 (0.992-1.037)
**FT cartilage loss**					
Tibiofemoral	0.986 (0.923-1.054)	2.193 (0.420-11.438)	0.979 (0.909-1.053)	3.020 (0.410-22.232)	1.129 (1.025-1.243
Patellofemoral	0.998 (0.961-1.035)	1.243 (0.491-3.145)	0.994 (0.957-1.033)	1.384 (0.522-3.668)	1.068 (0.983-1.161)
Any	1.000 (0.967-1.034)	1.406 (0.602-3.283)	0.996 (0.962-1.032)	1.590 (0.638-3.964)	1.074 (1.008-1.144)
**Size of BML**					
Tibiofemoral	0.999 (0.939-1.062)	1.819 (0.427-7.760)	0.990 (0.926-1.059)	2.964 (0.556-15.797)	1.180 (1.077-1.293)
Patellofemoral	1.017 (0.978-1.057)	1.086 (0.373-3.167)	1.016 (0.976-1.057)	1.109 (0.390-3.156)	1.018 (0.910-1.140)
Any	1..009 (0.976-1.044)	1.462 (0.625-3.418)	1.006 (0.971-1.042)	1.636 (0.693-3.858)	1.069 (0.987-1.159)
**% that is BML**					
Tibiofemoral	1.007 (0.938-1.082)	2.040 (0.390-10.671)	1.000 (0.925-1.080)	2.751 (0.403-18.782)	1.129 (1.045-1.219)
Patellofemoral	1.010 (0.965-1.058)	0.938 (0.298-2.948)	1.009 (0.962-1.058)	0.991 (0.303-3.241)	1.044 (0.960-1.135)
Any	1.009 (0.970-1.050)	1.346 (0.527-3.440)	1.006 (0.965-1.048)	1.501 (0.556-4.052)	1.069 (1.010-1.132)
**Osteophytes**					
Tibial medial	0.918 (0.855-0.985)	4.508 (1.020-19.926)	0.905 (0.836-0.979)	9.854 (1.503-64.617)	1.185 (1.116-1.258)
Tibial lateral	0.956 (0.901-1.014)	2.921 (0.718-11.887)	0.947 (0.887-1.010)	4.579 (0.836-25.087)	1.146 (1.061-1.237)
Femoral medial	0.935 (0.869-1.005)	4.206 (0.899-19.676)	0.921 (0.849-1.000)	9.163 (11.302-64.476)	1.190 (1.116-1.269)
Femoral lateral	0.946 (0.900-0.995)	2.932 (1.009-8.515)	0.938 (0.890-0.989)	4.208 (1.188-14.907)	1.124 (1.067-1.184)
Tibiofemoral	0.947 (0.908-0.987)	3.300 (1.323-8.229)	0.939 (0.899-0.982)	4.615 (1.623-13.124)	1.115 (1.066-1.166)

Patellofemoral	0.996 (0.978-1.014)	1.517 (1.032-2.230)	0.994 (0.976-1.012)	1.608 (1.079-2.397)	1.039 (1.010-1.069)
Any	0.994 (0.977-1.011)	1.558 (1.085-2.239)	0.992 (0.975-1.009)	1.666 (1.141-2.431)	1.044 (1.017-1.071)
**Other**					
Patellar tend. signal*	0.981 (0.903-1.065)	0.872 (0.132-5.766)	0.979 (0.901-1.064)	0.819 (0.119-5.615)	1.046 (0.929-1.177)
Any ganglion cyst *	0.942 (0.904-0.983)	0.405 (0.159-1.031)	0.942 (0.903-0.983)	0.400 (0.156-1.025)	1.009 (0.947-1.075)
Inf.pat. bursa signal*	0.980 (0.939-1.023)	0.581 (0.220-1.534)	0.980 (0.939-1.023)	0.578 (0.218-1.534)	1.004 (0.937-1.075)
Prepat. bursa signal*	0.996 (0.963-1.030	1.232 (0.564-2.692)	0.994 (0.961-1.029)	1.188 (0.541-2.612)	1.036 (0.981-1.093
Popliteal cyst*	0.991 (0.959-1.024)	0.670 (0.316-1.420)	0.989 (0.958-1.022)	0.646 (0.303-1.377)	1.032 (0.979-1.088)
Joint effusion	1.019 (0.998-1.041)	1.140 (0.688 (1.891)	1.016 (0.995-1.037)	1.254 (0.759-2.074)	1.066 (1.038-1.095)
Hoffa-synovitis	0.997 (0.983-1.012)	1.166 (0.831-1.635)	0.996 (0.982-1.011)	1.188 (0.843-1.675)	1.016 (0.993-1.040)

Figures S1. Flow chart representation the study population and the analyses performed., NFBC1986, Northern Finland Birth Cohort 1986; Hb, hemoglobin; MRI, magnetic resonance imaging; OA, osteoarthritis; BMI, body mass index; fP, fasted plasma; LDL, low-density lipoprotein; hs-CRP, high-sensitivity C-reactive protein; BML, bone marrow lesion; FT, full thickness; ACL, anterior cruciate ligament; PCL, posterior cruciate ligament.

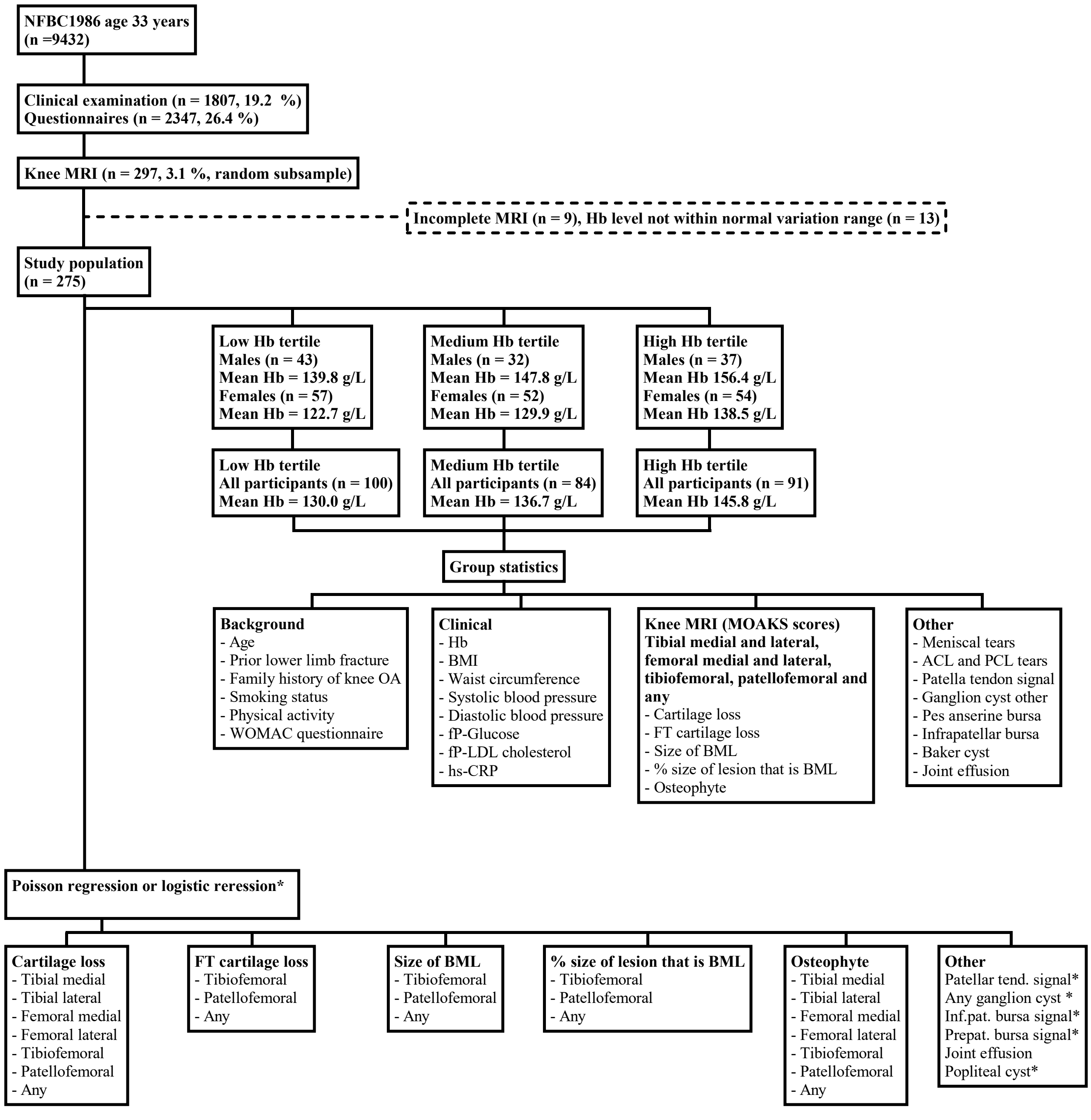

Supplement: Multimedia component 1 [file mmc1.pdf]
